# Comparing the Infectivity of Recent SARS-CoV-2 Omicron Sub-Variants in Syrian Hamsters

**DOI:** 10.3390/v16010122

**Published:** 2024-01-14

**Authors:** Rana Abdelnabi, Ria Lassaunière, Piet Maes, Birgit Weynand, Johan Neyts

**Affiliations:** 1 Department of Microbiology, Immunology and Transplantation, Laboratory of Virology and Chemotherapy, Rega Institute, KU Leuven, B-3000 Leuven, Belgium; rana.abdelnabi@kuleuven.be; 2 Department of Microbiology, Immunology and Transplantation, VirusBank Platform, KU Leuven, B-3001 Leuven, Belgium; 3 Statens Serum Institut, DK-2300 Copenhagen, Denmark; mlas@ssi.dk; 4 Department of Microbiology, Immunology and Transplantation, Laboratory of Clinical and Epidemiological Virology, Rega Institute, KU Leuven, B-3000 Leuven, Belgium; 5 Department of Imaging and Pathology, Translational Cell and Tissue Research, Division of Translational Cell and Tissue Research, KU Leuven, B-3000 Leuven, Belgium; birgit.weynand@uzleuven.be

**Keywords:** COVID-19, SARS-CoV-2, Omicron, hamsters, infectivity, EG5.1, BA.2.86

## Abstract

Since the emergence of the first omicron SARS-CoV-2 variant at the end of 2021, several sub-variants have evolved and become predominant in the human population, showing enhanced transmissibility and ability to (partly) escape the adaptive immune response. The XBB sub-variants (e.g., EG.5.1) have become globally dominant. Besides the XBB sub-variants, a phylogenetically distinct variant, i.e., BA.2.86, is also circulating; it carries several mutations in the spike protein as compared to its parental BA.2 variant. Here, we explored the infectivity of the BA.2.86 and EG.5.1 sub-variants compared to the preceding BA.5 sub-variant in Syrian hamsters. Such preclinical models are important for the evaluation of updated vaccine candidates and novel therapeutic modalities. Following intranasal infection with either variant, throat swabs and lung samples were collected on days 3 and 4 post infection. No significant differences in viral RNA loads in throat swabs were observed between these sub-variants. However, the infectious virus titers in the lungs of EG.5.1- and BA.2.86-infected animals were significantly lower compared to the BA.5-infected ones. The lung pathology scores of animals infected with EG.5.1 and BA.2.86 were also markedly lower than that of BA.5 sub-variant. Together, we show that EG.5.1 and BA.2.86 sub-variants exhibit an attenuated replication in hamsters’ lungs as compared to the BA.5 sub-variant.

## 1. Introduction

In the first few months after its emergence, the Severe Acute Respiratory Syndrome Coronavirus 2 (SARS-CoV-2) was initially predicted to possess a slow genetic diversification due to the proofreading function of the nonstructural protein 14 (nsp14) exoribonuclease [1]. However, following the massive global spread of the virus infection, SARS-CoV-2 has shown an intriguing evolutionary adaptation that often led to an increased evasion of natural immunity and vaccine-acquired immunity [2]. A remarkable milestone in SARS-CoV-2 genetic evolution was the emergence of the first Omicron (B.1.1.529) variant in November 2021 [3]. The omicron variant showed a significant enhancement of transmissibility compared to the previous variants of concerns [4] but with a lower risk of hospitalization and death [5]. It has been suggested that the enhanced infectivity of the omicron variant could be attributed to an enhanced binding of the viral spike protein with Neuropilin-1 (Nrp1), a cell surface receptor that regulates multiple vital biological processes [6]. Following that, numerous Omicron sub-lineages (sub-variants) have emerged and become predominant in the human population [4]. SARS-CoV-2 XBB descendants, such as XBB.1.5 and EG.5.1 have become globally dominant. In the summer of 2023, the hyper-mutated BA.2.86 variant emerged; it has >40 new mutations, of which 34 in the spike protein, as compared to its BA.2 ancestor [7,8]. Some of these mutations are assumed to be associated with immune evasion and escape from some monoclonal antibodies (MAbs) [7,8]. With an extra mutation in the spike protein, namely, L455S mutation, BA.2.86 evolved to another subvariant, i.e., JN.1, which is globally rapidly spreading in December 2023 [9].

Here, we investigate the infectivity of the currently circulating BA.2.86 and EG.5.1 sub-variants in comparison with a preceding BA.2 descendant, namely the BA.5 sub-variant in Syrian hamsters. Our aim is to assess the possibility of establishing a robust preclinical model for the evaluation of the efficacy of new vaccine and therapeutic agents (especially monoclonal antibodies) against these two recent variants.

## 2. Materials and Methods

### 2.1. Viruses

The omicron SARS-CoV-2 variants used in this study are: BA.5 sub-variant (BA.5.2.1, EPI_ISL_14782497, passage 3 stock), BA.2.86 variant (SARS-CoV-2/hu/DK/SSI-H135, passage 2 stock), and EG.5.1 variant (SARS-CoV-2/hu/DK/SSI-H121, passage 2 stock). the whole-genome sequences of the BA.2.86 and EG.5.1 variants are available in the European Nucleotide Archive under the project number PRJEB67449 with accession numbers OY747653 and OY747654, respectively. Live virus-related work was conducted in the high-containment A3 and BSL3+ facilities of the KU Leuven Rega Institute (3CAPS) under licenses AMV 30112018 SBB 219 2018 0892 and AMV 23102017 SBB 219 20170589 according to institutional guidelines.

### 2.2. SARS-CoV-2 Infection Model in Hamsters

**Figure 1:**
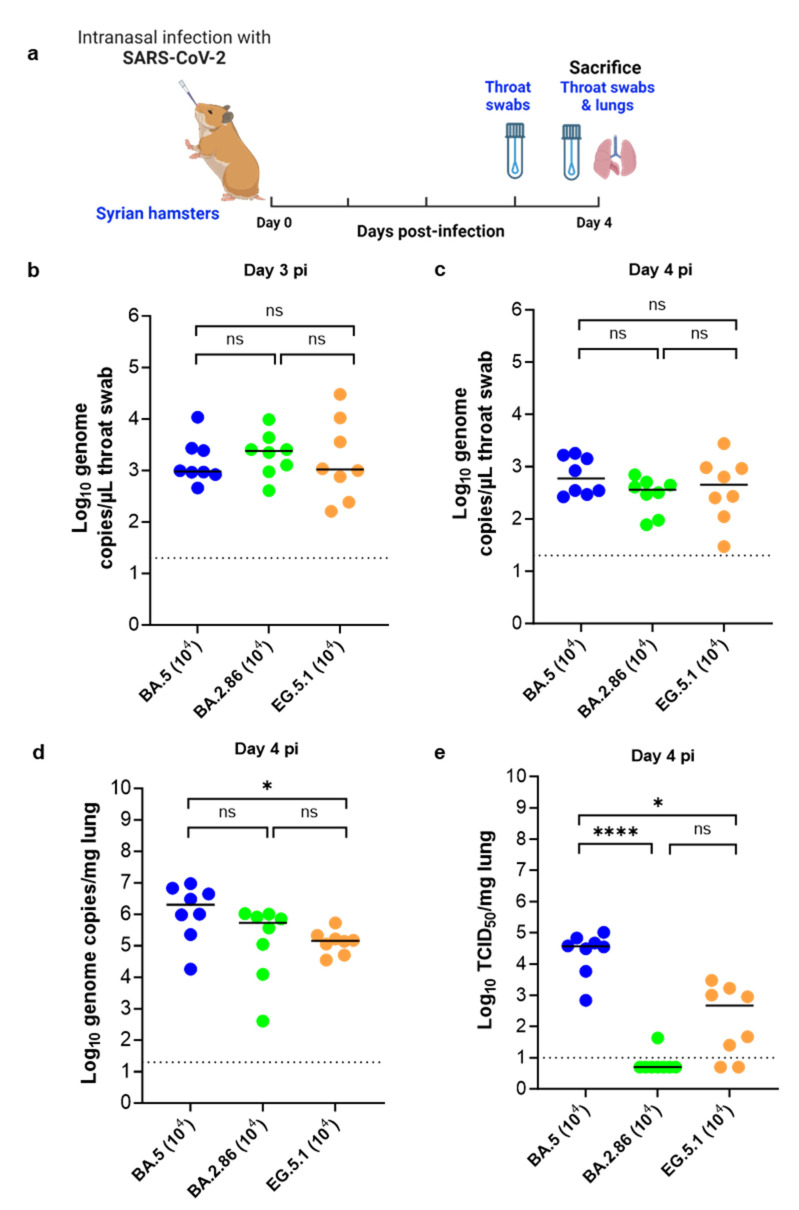
Viral loads in the throat swabs and lungs of Syrian hamsters following infection with different omicron SARS-CoV-2 variants. (**a**) Set-up of the Syrian hamster infection study. (**b**,**c**) Viral RNA levels in the throat swabs of hamsters infected with 10^4^ TCID_50_ of BA.5, BA.2.86 or EG.5.1 omicron SARS-CoV-2 variants on day 3 and day 4 post infection (pi), respectively, are expressed as log_10_ SARS-CoV-2 RNA copies per µL throat swab. (**d**) Viral RNA levels in the lungs of infected hamsters on day 4 pi are expressed as log_10_ SARS-CoV-2 RNA copies per mg lung tissue. Individual data and median values are presented. (**e**) Infectious viral loads in the lungs of infected hamsters on day 4 pi are expressed as log_10_ TCID_50_ per mg lung tissue. Individual data and median values are presented. Data were analyzed with the Kruskal–Wallis test, * *p* < 0.05, **** *p* <0.0001, ns = non-significant. Dotted lines represent limit of quantification. All data are from 2 independent experiments with n = 8 per group.

The hamster infection model of SARS-CoV-2 has been described before [10,11]. In brief, 6–8-week-old female Syrian hamsters were intranasally infected with 100 µL containing approximately 10^4^ TCID_50_ of the selected SARS-CoV-2 omicron variant (Figure 1a). Throat swabs were collected on day 3 and day 4 post infection (pi) using Urethral swabs with mini tips (COPAN Diagnostics, Murrieta, CA, USA). At day 4 pi, animals (n = 8 per variant) were euthanized by i.p. injection of 500 μL Dolethal (200 mg/mL sodium pentobarbital, Vetoquinol, Niel, Belgium) for sampling of the lungs and further analysis [12]. Housing conditions and experimental procedures were approved by the ethics committee of animal experimentation of KU Leuven (license P065-2020). A smaller group of animals (n = 4) were also euthanized for each variant on day 3 pi for lung collection.

### 2.3. SARS-CoV-2 RT-qPCR

Hamster lung tissues were collected after sacrifice and were homogenized using bead disruption (Precellys tubes, Bertin Corp., Rockville, MD, USA) in TRK lysis buffer (E.Z.N.A.^®^ Total RNA Kit, Omega Bio-tek, Norcross, GA, USA) and centrifuged (10,000 rpm, 5 min) to pellet the cell debris. RNA was extracted according to the manufacturer’s instructions. Of 50 μL eluate, 4 μL was used as a template in RT-qPCR reactions. RT-qPCR was performed on a LightCycler96 platform (Roche Diagnostics, Diegem, Belgium) using the iTaq Universal Probes One-Step RT-qPCR kit (BioRad, Temse, Belgium) with N2 primers and probes targeting the nucleocapsid [10,11]. Standards of SARS-CoV-2 cDNA (IDT) were used to express viral genome copies per mg tissue or per mL serum.

### 2.4. Endpoint Virus Titrations

Lung tissues were homogenized using bead disruption (Precellys) in 350 µL minimal essential medium and centrifuged (10,000 rpm, 5 min, 4 °C) to pellet the cell debris. To quantify infectious SARS-CoV-2 particles, endpoint titrations were performed on confluent Vero E6 cells in 96-well plates. Viral titers were calculated via the Reed and Muench method [13] using the Lindenbach calculator and were expressed as 50% tissue culture infectious dose (TCID_50_) per mg tissue.

### 2.5. Histopathology

For histological examination, the lungs were fixed overnight in 4% formaldehyde (diluted from Pierce™ 16% Formaldehyde (*w*/*v*), Thermo Scientific, Dilbeek, Belgium) and embedded in paraffin. Tissue sections (5 μm) were analyzed after staining with hematoxylin and eosin and scored blindly for lung damage by an expert pathologist. The scored parameters, to which a cumulative score of 1 to 3 was attributed, were the following: congestion, intra-alveolar hemorrhagic, apoptotic bodies in bronchus wall, necrotizing bronchiolitis, perivascular edema, bronchopneumonia, perivascular inflammation, peribronchial inflammation, and vasculitis.

### 2.6. Group Size Justification

To calculate our sample size, we used Gpower statistical software with the following parameters: type = F test, statistical test = ANOVA, fixed effects, omnibus, one way, power = 0.9, an effect size of 0.8, alfa = 0.05, and number of groups = 3. This indicated that we need at least 8 animals per group.

### 2.7. Statistics

GraphPad Prism 10 (GraphPad Software, Inc., San Diego, CA, USA) was used to perform statistical analysis. Statistical significance was determined using the non-parametric Kruskal–Wallis test. *p*-values of <0.05 were considered significant.

## 3. Results and Discussion

In this study, we aimed to investigate the infectivity of the two recent and important omicron sub-variants BA.2.86 and EG.5.1 in comparison with the earlier BA.2 descendant sub-variant (BA.5) in Syrian hamsters. The BA.2 variant was not included in this study as a comparator as it does not replicate efficiently in our hamster model as compared to the BA.5 sub-variant. In brief, the animals were intranasally infected with 1 × 10^4^ TCID_50_ of either variants, throat swabs were collected on days 3 and 4 post infection (pi), and then animals were sacrificed on day 4 pi for the collection of lung tissues (Figure 1a). A small group of animals were also sacrificed on day 3 pi to obtain data on infectious viral loads in the lungs on that day pi (Supplementary Figure S1).

No significant differences in viral RNA loads were observed in the throat swabs samples collected from the three variants groups either on day 3 (Figure 1b) or day 4 (Figure 1c) pi. In general, the viral RNA loads of the three variants in the throat swabs samples were higher on day 3 pi compared to day 4 pi (Figure 1b). Median viral RNA loads in the lungs on day 4 pi were 2.1 × 10^6^, 5.4 × 10^5^, and 1.4 × 10^5^ genome copies/mg tissue for the groups infected with BA.5, BA.2.86, and EG.5.1 (*p* = 0.029 compared to BA.5 group, Kruskal–Wallis), respectively (Figure 1d). The highest infectious virus titer in the lungs on day 4 pi was observed in the BA.5-infected group with a median titer of around 3.6 × 10^4^ TCID_50_/mg of lung tissue (Figure 1d). The EG.5.1-infected group showed a median titer of 4.6 × 10^2^ TCID_50_/mg of lung tissue (*p* = 0.049 compared to BA.5 group, Kruskal–Wallis) (Figure 1d). Strikingly, no infectious virus titers were detected in the lungs of 11 out of 12 animals infected with the BA.2.86 variant (*p* = 0.0001 compared to BA.5 group, Kruskal–Wallis) (Figure 1d). A similar pattern of infectious virus titers in the lungs was also observed on day 3 pi (Supplementary Figure S1).

**Figure 2:**
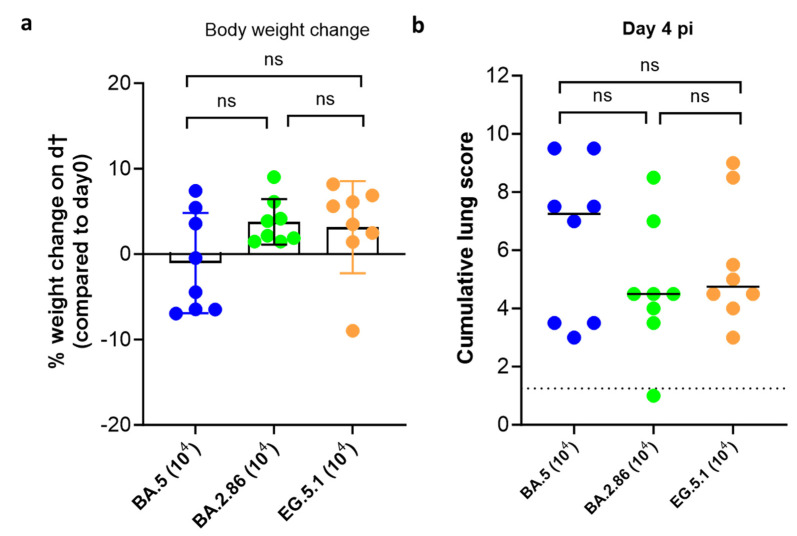
Body weight change and lung pathology of Syrian hamsters following infection with different omicron SARS-CoV-2 variants. (**a**) Weight change of hamsters infected with 10^4^ TCID_50_ of BA.5, BA.2.86, or EG.5.1 omicron SARS-CoV-2 variants at day 4 post infection (pi) in percentage, normalized to the body weight at the time of infection. Bars represent means ± SD. (**b**) Cumulative severity score from H&E-stained slides of lungs from infected hamsters on day 4 pi. Individual data and median values are presented and the dotted line represents the median score of untreated non-infected hamsters. Data were analyzed with the Kruskal–Wallis test; ns = non-significant. Dotted line represents the average score for non-infected animals. All data are from 2 independent experiments with n = 8 per group.

Although no significant differences in body weight change (on day 4 compared to day 0) was noted between the three variants groups (Figure 2a), the group infected with BA.5 presented with the lowest body weight gain profile (with average weight change of -1%). The average % body weight change for groups infected with BA.2.86 and EG.5.1 were 3.8 and 3.1%, respectively (Figure 2a). Moreover, the median cumulative lung pathology scores for BA.2.86 (median score of 4.5)- and EG.5.1 (median score of 4.75)-infected animals were markedly lower as compared to the scores in animals infected with BA.5 (median score of 7.25) (Figure 2b, Table 1). A recent study revealed that BA.2.86-infected hamsters in general has significantly lower viral RNA loads in oral swabs and lungs compared to EG.5.1-infected hamsters on day 2 and 5 pi. No infectious virus loads were quantified in that study [14]. However, in our model, the only remarkable difference between these two sub-variants was in the infectious virus loads in the lungs of the infected animals (both on days 3 and 4 pi).

## 4. Conclusions

We show here that both EG.5.1 and BA.2.86 sub-variants are attenuated in Syrian hamsters as compared to the other BA.2 descendant sub-variant (BA.5), especially in terms of infectious virus titers in the lungs. Similar to the situation in humans, our data show that the recently emerged omicron sub-variants, so far, do not have higher virulence in vivo compared to the earlier sub-variants. However, the continuous emergence of such sub-variants will remain a challenge as it may further require updating vaccines and therapeutic antibodies. Pre-clinical models with the evolving sub-variants are therefore crucial not only to study the virological characteristics of these new sub-variants, but more importantly to allow us to evaluate the efficacy of updated and also novel vaccines as well as therapeutic options for which the efficacy is virus variant dependent, which is mostly neutralizing antibodies. The main limitation of our study is that we could not detect any infectious virus titers in the lungs of the hamsters infected with the BA.2.86 sub-variant although we detected rather high viral RNA loads. Therefore, viral RNA loads in the throat swabs and the lungs as well as lung histopathology scores could be a more suitable readout for vaccine and antiviral studies involving these two new sub-variants.

## Figures and Tables

**Table 1:** Detailed lung histopathology scoring for Syrian hamsters infected with different omicron sub-variants on day 4 post infection (pi).

Group	Hamster	Congestion	Intra-Alveolar Hemorrhage	Intra-Alveolar Edema	Apoptotic Bodies in Bronchus Wall	Perivascular Edema	Bronchopneumonia	Perivascular Inflammation	Peribronchial Inflammation	Vasculitis	Cumulative Score
BA.5 (d4 pi)	1	1	1	1	1		1	1	0.5	0.5	7
	2	1			1			0.5	1		3.5
	3	1			1			0.5	1		3.5
	4	1	1					0.5	0.5		3
	5	1		1		1	1	2	0.5	1	7.5
	6	1		1	1		1	2	0.5	1	7.5
	7	1		1	1	1	2	2	0.5	1	9.5
	8	1		1	1	1	2	2	0.5	1	9.5
BA.2.86 (d4 pi)	9	1	1		1			0.5	0.5		4
	10	1			1		0.5	1	0.5	0.5	4.5
	11	1			1		1	1	0.5		4.5
	12	1									1
	13	1		1	1	1	1	2	0.5	1	8.5
	14	1			1			0.5	1		3.5
	15	1			1		0.5	1	0.5	0.5	4.5
	16	1		1		1	1	2	0.5	0.5	7
EG.5.1 (d4 pi)	17	1	1		1		1	1	0.5		5.5
	18	1			1			0.5	0.5		3
	19	1	1		1			0.5	1	0.5	5
	20	1	1		1			0.5	1		4.5
	21	1					1	2	0.5		4.5
	22	1		1	1	1	1	2	1	0.5	8.5
	23	1	1	1		1	2	2		1	9
	24	1	1		1			0.5	0.5	0.5	4.5

## Data Availability

All of the data generated or analyzed during this study are included in the figures of this published article. Raw data files are available upon request to the corresponding author.

## Supplementary Materials

The following supporting information can be downloaded at: https://www.mdpi.com/article/10.3390/v16010122/s1, Figure S1: Viral loads in the lungs of Syrian hamsters following infection with different omicron SARS-CoV-2 variants on day 3 post infection. (a) Viral RNA levels in the lungs of hamsters infected with 10^4^ TCID_50_ of BA.5, BA.2.86, or EG.5.1 omicron SARS-CoV-2 variants on day 3 post infection (pi) are expressed as log_10_ SARS-CoV-2 RNA copies per mg lung tissue. Individual data and median values are presented. (b) Infectious viral loads in the lungs of infected hamsters on day 3 pi are expressed as log_10_ TCID_50_ per mg lung tissue. Individual data and median values are presented. Data were analyzed with the Kruskal–Wallis test; * *p* < 0.05; ns = non-significant. The data are from one experiment with n = 4 per group.
