# Supplementary material for: Comparing the Infectivity of Recent SARS-CoV-2 Omicron Sub-Variants in Syrian Hamsters
Source: Viruses. 2024 Jan 14;16(1):122. doi: 10.3390/v16010122 (PMC10819014; doi:10.3390/v16010122)
Supplement: Supplementary file 1 [file viruses-16-00122-s001.zip › viruses-2805237-supplementary.pdf]

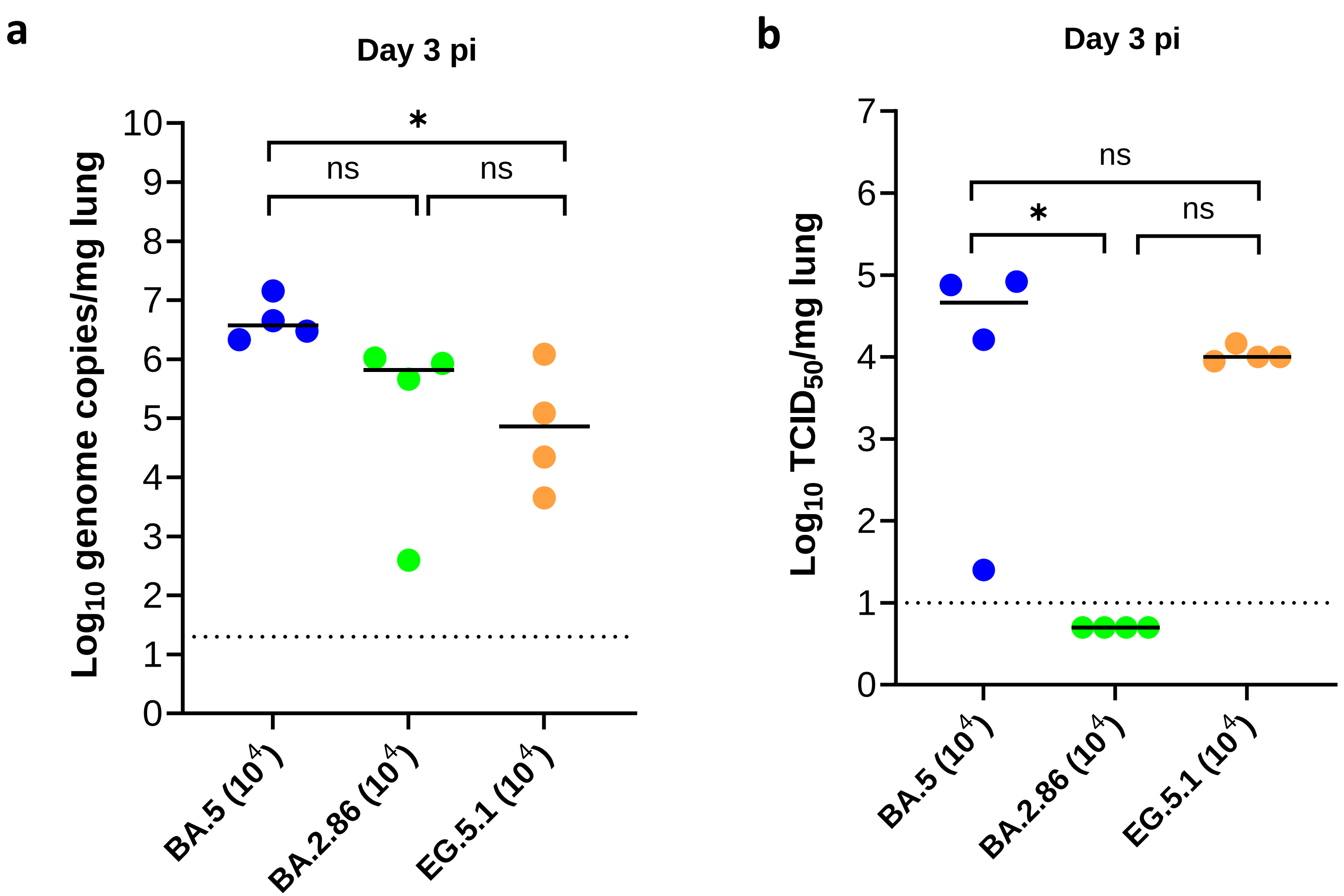

Figure S1: Viral loads in the lungs of Syrian hamsters following infection with different omicron SARS-CoV-2 variants on day 3 post-infection. (a) Viral RNA levels in the lungs of hamsters infected with 10^4 $TCID_{50}$ of BA.5, BA.2.86 or EG.5.1 omicron SARS-CoV-2 variants on day 3 post-infection (pi) are expressed as log_{10} SARS-CoV-2 RNA copies per mg lung tissue. Individual data and median values are presented. (b) Infectious viral loads in the lungs of infected hamsters on day 3 pi are expressed as log_{10} $TCID_{50}$ per mg lung tissue. Individual data and median values are presented. Data were analyzed with the Kruskal-Wallis test, *P<0.05, ns=non-significant. The data are from one experiment with n=4 per group.
